# Clinically relevant variants in a large cohort of Indian patients with Marfan syndrome and related disorders identified by next-generation sequencing

**DOI:** 10.1038/s41598-020-80755-7

**Published:** 2021-01-12

**Authors:** Shalini S. Nayak, Pauline E. Schneeberger, Siddaramappa J. Patil, Karegowda M. Arun, Pujar V. Suresh, Viralam S. Kiran, Sateesh Siddaiah, Shreesha Maiya, Shrikanth K. Venkatachalagupta, Neethukrishna Kausthubham, Fanny Kortüm, Isabella Rau, Alexandra Wey-Fabrizius, Lotte Van Den Heuvel, Josephina Meester, Lut Van Laer, Anju Shukla, Bart Loeys, Katta M. Girisha, Kerstin Kutsche

**Affiliations:** 1 Department of Medical Genetics, Kasturba Medical College, Manipal Academy of Higher Education, Manipal, India; 2 Institute of Human Genetics, University Medical Center Hamburg-Eppendorf, Martinistraße 52, 20246 Hamburg, Germany; 3 Narayana Hrudayalaya Hospitals/Mazumdar-Shaw Medical Center, Bangalore, India; 4 Center of Medical Genetics, University of Antwerp and Antwerp University Hospital, Antwerp, Belgium

**Keywords:** Genotype, Genetics, Clinical genetics, Disease genetics, Cardiovascular diseases

## Abstract

Marfan syndrome and related disorders are a group of heritable connective tissue disorders and share many clinical features that involve cardiovascular, skeletal, craniofacial, ocular, and cutaneous abnormalities. The majority of affected individuals have aortopathies associated with early mortality and morbidity. Implementation of targeted gene panel next-generation sequencing in these individuals is a powerful tool to obtain a genetic diagnosis. Here, we report on clinical and genetic spectrum of 53 families from India with a total of 83 patients who had a clinical diagnosis suggestive of Marfan syndrome or related disorders. We obtained a molecular diagnosis in 45/53 (85%) index patients, in which 36/53 (68%) had rare variants in *FBN1* (Marfan syndrome; 63 patients in total), seven (13.3%) in *TGFBR1*/*TGFBR2* (Loeys–Dietz syndrome; nine patients in total) and two patients (3.7%) in *SKI* (Shprintzen–Goldberg syndrome). 21 of 41 rare variants (51.2%) were novel. We did not detect a disease-associated variant in 8 (15%) index patients, and none of them met the Ghent Marfan diagnostic criteria. We found the homozygous *FBN1* variant p.(Arg954His) in a boy with typical features of Marfan syndrome. Our study is the first reporting on the spectrum of variants in *FBN1, TGFBR1, TGFBR2*, and *SKI* in Indian individuals.

## Introduction

Heritable connective tissue disorders (HCTD) comprise a group of multisystem diseases affecting the heart, blood vessels, bone, eyes, skin, joints, and lungs. Marfan syndrome (MFS, MIM#154700), Loeys–Dietz syndrome (LDS, MIM#609192, MIM#610168, MIM#613795, MIM#614816, MIM#615582), and Shprintzen–Goldberg syndrome (SGS, MIM#182212) belong to the HCTDs and share many clinical features, such as cardiovascular, skeletal, craniofacial, ocular, and cutaneous abnormalities^1^. The phenotype of MFS is characterized by aortic root aneurysm or dissection, mitral valve prolapse, ectopia lentis, long bone overgrowth, joint laxity, and skin striae as the key abnormalities. Craniofacial dysmorphism includes dolichocephaly, exophthalmos, downslanted palpebral fissures, malar hypoplasia, highly arched palate, and micro- or retrognathia^2^. Heterozygous pathogenic variants in the *FBN1* gene, encoding the extracellular matrix protein fibrillin-1, are the cause of MFS^3^. Pathogenic *FBN1* variants are spread over the entire gene and comprise sequence-level alterations, such as missense, nonsense, frameshift, and splice variants, identified in the majority of MFS-affected cases as well as single- and multi-exon deletions in up to 5% of the affected individuals^2,4^. LDS has many clinical manifestations in common with MFS, however, LDS-affected patients can have characteristic craniofacial features, such as hypertelorism, abnormal uvula or cleft palate^5^. Typical cardiovascular features in LDS are dilatation of the aortic root at the level of the sinus of Valsalva, aneurysms affecting thoracic and abdominal aorta and arterial branches, as well as arterial tortuosity^1,5^. Cardiovascular manifestations tend to be more severe in LDS than in MFS^6^, however, a multi-center study has recently demonstrated a comparable cardiovascular outcome in individuals with MFS and LDS^7^. Heterozygous pathogenic variants in six genes cause LDS type 1–6: *TGFBR1*, *TGFBR2*, *SMAD3*, *TGFB2*, *TGFB3*, and *SMAD2*^8–13^. Patients with SGS have some of the craniofacial, skeletal, skin and cardiovascular manifestations of MFS and LDS, but in addition show intellectual disability, skeletal muscle hypotonia and craniosynostosis. Mitral valve prolapse and aortic root dilatation have been reported in some cases^14^. In SGS-affected probands mainly de novo heterozygous pathogenic variants in the *SKI* gene have been identified that cluster in two regions, one encoding the R-SMAD binding domain and the other encoding the Dachshund-homology domain^15–17^.

Implementation of targeted gene panel next-generation sequencing (NGS) in individuals with HCTD or hereditary aortopathies in a clinical setting has been proven to be powerful in obtaining a genetic diagnosis: a pathogenic or likely pathogenic variant was identified in 3.9–35.5% of the patients tested in different centers worldwide^18–26^. Thus, in individuals with clinical features typical of HCTD or with a non-syndromic form of aortopathy an NGS-based molecular test is the most practical screening method to identify the disease-related sequence variant. Here, we report on our clinical and genetic findings after testing of 53 index patients from India with a clinical diagnosis suggestive of HCTD using targeted NGS and whole-exome sequencing. Although our patient cohort is small, this is the first study reporting on the spectrum of variants in *FBN1*, *TGFBR1*, *TGFBR2*, and *SKI* in Indian individuals, with about 50% novel pathogenic variants.

## Results

We recruited 83 patients from 53 families with MFS, aortopathy or a related HCTD. The ages of the patients ranged from 3 months to 56 years with a median age of 14 years. The majority were males (51, 61.5%; CI 95% 51–71) and children and adolescents (53, 64% were less than 18 years of age; CI 95% 53–73). Twenty-one families (39% of 53 families; CI 95% 28–53) had more than one affected individual, including a set of monozygotic twins. Echocardiographic information was available for 77/83 individuals that included all index patients. Ophthalmological, skeletal and other information were available for 72/83 individuals.

### Molecular findings in 53 unrelated Indian patients and their family members with HCTD

**Figure 1:**
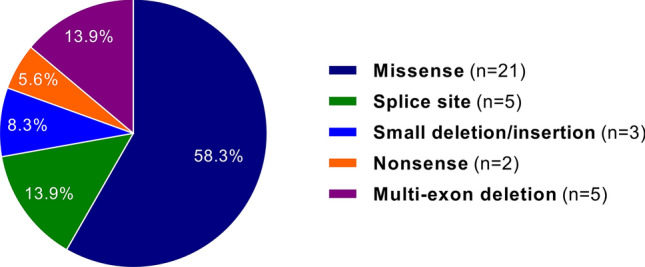
A representation of the different *FBN1* variants in 36 index patients is shown.

NGS-based genetic testing was performed in 53 unrelated Indian individuals with MFS, aortopathy or a related HCTD. Of the 53 individuals, 44 (83.0%; CI 95% 71–91) tested positive for a pathogenic variant in *FBN1*, *TGFBR1*, *TGFBR2* or *SKI*, and 1 (1.9%; CI 95% 0.3–10) had a variant of unknown significance (VUS) in *FBN1* (Table 1 and Supplementary Table S1). 36 of the 45 (80.0%; CI 95% 66–89) patients with a rare variant carried an *FBN1* variant, including 21 missense (58.3%; CI 95% 42–73), five splice (13.9%; CI 95% 6–29), three small deletion/insertion (8.3%; CI 95% 3–22), two nonsense (5.6%; CI 95% 2–18), and five multi-exon deletions (13.9%; CI 95% 6–29) (Fig. 1). The known pathogenic *FBN1* missense variant c.2861G > A/p.(Arg954His)^27^ was identified in patient 10 in a homozygous state. Both parents (first cousins) were heterozygous carriers of this *FBN1* alteration. 4 (8.9%; CI 95% 4–21) patients had a *TGFBR1* missense variant, 3 (6.7%; CI 95% 2–18) a *TGFBR2* missense variant, and 2 (4.4%; CI 95% 1–15) a *SKI* missense variant. The *FBN1* variants c.(1468 + 1_1469-1)_(1837 + 1_1838-1)del, c.3037G > A/p.(Gly1013Arg), and c.7828G > A/p.(Glu2610Lys) and the *TGFBR1* variant c.722C > T/p.(Ser241Leu) have been identified in two non-consanguineous families each. Out of the 41 different rare variants in four genes, 20 (48.8%; CI 95% 34–63) have been previously reported in the HGMD professional and/or UMD-FBN1 database and 21 (51.2%; CI 95% 36–66) were novel (Table 1 and Supplementary Table S1). 20 of the 21 novel variants were classified as pathogenic or likely pathogenic, and the intronic *FBN1* variant c.2419 + 3delinsTTTTAGATCCATATTTTAG (in family 9) was interpreted as VUS (Table 1). In 17 (37.8%; CI 95% 25–52) index patients, de novo occurrence of the pathogenic variant [ten known and seven novel variants (Table 1 and Supplementary Table S1)] was confirmed by genetic testing of the healthy parents (without confirming paternity), including 11 variants in *FBN1*, 3 in *TGFBR1*, 2 in *TGFBR2*, and 1 in *SKI*. Segregation analysis was performed in 21 families with a minimum of two affected individuals, and 29 relatives were found to carry the familial variant, including 27 individuals with an *FBN1* variant. NGS of 62 genes/candidate genes related to HCTD and hereditary aortopathies (single nucleotide variant and copy number variation analysis) and MLPA analysis (all exons of *FBN1*, *TGFBR1*, *TGFBR2*, and 15 selected exons of *COL3A1;* see material and methods section for details) did not detect a disease-associated variant in 8 (15%; CI 95% 8–27) index patients.

**Table 1:** Number of affected family members, in silico pathogenicity predictions and ACMG classification for novel variants found in the cohort.

Gene	Patient #	Affected family members	Nucleotide change	Affected exon(s)/intron(s)	Amino acid alteration	gnomAD MAF [%]	Pathogenicity predictions			Splice predictions	ACMG	
							CADD	REVEL	M-CAP		Classification	Criteria
***FBN1***^**c**^	1	1	c.(?_1317)_(1837 + 1_1838-1)del	Upstream of exon 1 and exons 1–15	p.?	NA	NA	NA	NA	Not done	LP^#^	PVS1, PM2
	3	1 (de novo)^a^	c.1130G > A	Exon 10	p.(Cys377Tyr)	Absent	33	0.927	0.915	Not done	LP	PM1, PM2, PM5, PM6, PP2, PP3, PP5
	4	2	c.1463G > A	Exon 12	p.(Cys488Thr)	Absent	32	0.937	0.892	Not done	LP	PM1, PM2, PM5, PP2, PP3, PP5
	5^b^	2	c.(1468 + 1_1469-1)_(1837 + 1_1838-1)del	Exons 13–15	p.?	NA	NA	NA	NA	Not done	P^#^	PVS1, PM2, PS4
	6^b^	3	c.(1468 + 1_1469-1)_(1837 + 1_1838-1)del	Exons 13–15	p.?	NA	NA	NA	NA	Not done	P^#^	PVS1, PM2, PS4, PP1
	8	2	c.1867T > A	Exon 16	p.(Cys623Ser)	Absent	27.6	0.771	0.493	Not done	LP	PM1, PM2, PM5, PP2, PP3
	9	2	c.2419 + 3delinsTTTTAGATCCATATTTTAG	Intron 20	p.?	Absent	14.96	NA	NA	Impact on splicing	VUS^#^	PM2, PP3
	14	1 (de novo)^a^	c.3589 + 1G > A	Intron 29	p.?	Absent	34	NA	NA	Impact on splicing	P	PVS1, PM2, PM6, PP3
	16	1	c.3635G > A	Exon 30	p.(Cys1212Tyr)	Absent	32	0.965	0.899	Not done	LP	PM1, PM2, PM5, PP2, PP3
	18	2	c.4491C > G	Exon 37	p.(Cys1497Trp)	Absent	17.95	0.768	0.820	Not done	LP	PM1, PM2, PM5, PP2, PP3
	21	2	c.4817-1_4819delGATA	Intron 39/exon 40	p.?	Absent	37	NA	NA	Impact on splicing	P	PVS1, PM2, PP3
	22	1 (de novo)^a^	c.5467_5474dupGAATGCAT	Exon 45	p.(Ile1825Metfs*71)	Absent	33	NA	NA	Not done	P	PVS1, PM2, PM6, PP3
	25	3	c.5621G > T	Exon 46	p.(Cys1874Phe)	Absent	33	0.991	0.981	Not done	LP	PM1, PM2, PP1, PP2, PP3
	27	1 (de novo)^a^	c.5671 + 1G > C	Intron 46	p.?	Absent	33	NA	NA	Impact on splicing	P	PVS1, PM2, PM6, PP3
	28	1 (de novo)^a^	c.5917 + 1G > T	Intron 48	p.?	Absent	34	NA	NA	Impact on splicing	P	PVS1, PM2, PM6, PP3, PP5
	29	1	c.5966G > C	Exon 49	p.(Cys1989Ser)	Absent	25.2	0.818	0.946	Not done	LP	PM1, PM2, PM5, PP2, PP3
	30	3	c.5993G > T	Exon 49	p.(Cys1998Phe)	Absent	33	0.956	0.928	Not done	LP	PM1, PM2, PM5, PP1, PP2, PP3
	32	2	c.(7204 + 1_7205-1)_(7819 + 1_7820-1)del	Exons 59–63	p.?	NA	NA	NA	NA	Not done	LP^#^	PVS1, PM2
	34	2	c.7817T > A	Exon 63	p.(Val2606Asp)	Absent	33	0.665	0.270	Not done	LP	PM1, PM2, PP2, PP3
***SKI***^**d**^	38	1 (de novo)^a^	c.104C > G	Exon 1	p.(Pro35Arg)	Absent	23.6	0.759	0.964	Not done	LP	PM1, PM2, PM5, PM6, PP3
***TGFBR2***^**e**^	43	1	c.1453C > A	Exon 6	p.(Arg485Ser)	Absent	29.4	0.868	0.374	Not done	LP	PM1, PM2, PM5, PP2, PP3
	44	1 (de novo)^a^	c.1454G > C	Exon 6	p.(Arg485Pro)	Absent	33	0.937	0.427	Not done	LP	PM1, PM2, PM5, PM6, PP2, PP3

The functional impact of the identified variants was predicted by the Combined Annotation Dependent Depletion (CADD) tool, the Rare Exome Variant Ensemble Learner (REVEL) scoring system, and the Mendelian Clinically Applicable Pathogenicity (M-CAP) Score. CADD is a framework that integrates multiple annotations in one metric by contrasting variants that survived natural selection with simulated mutations. Reported CADD scores are phred-like rank scores based on the rank of that variant’s score among all possible single nucleotide variants of hg19, with 10 corresponding to the top 10%, 20 at the top 1%, and 30 at the top 0.1%. The larger the score the more likely the variant has deleterious effects; the score range observed here is strongly supportive of pathogenicity, with all observed variants ranking above ~ 99% of all variants in a typical genome and scoring similarly to variants reported in ClinVar as pathogenic (~ 85% of which score > 15)^61^. REVEL is an ensemble method predicting the pathogenicity of missense variants with a strength for distinguishing pathogenic from rare neutral variants with a score ranging from 0 to 1. The higher the score the more likely the variant is pathogenic^62^. M-CAP is a classifier for rare missense variants in the human genome, which combines previous pathogenicity scores (including SIFT, Polyphen-2, and CADD), amino acid conservation features and computed scores trained on mutations linked to Mendelian diseases. The recommended pathogenicity threshold is > 0.025^63^. Splice site prediction scores were calculated for wild-type and mutated sequences by using the programs Human Splicing Finder 3.1, NetGene2, and the Berkeley Drosophila Genome Project Database^65–68^. Genetic tolerance at the affected amino acid position in the protein was predicted by MetaDome^64^. All variants were classified according to the guidelines of the American College of Medical Genetics (ACMG) either by use of an adjusted automated interpretation by VarSome (https://varsome.com/)^60^or in case of whole exon deletions and Indels (#) by manual application of the guidelines.

*LP* likely pathogenic, *NA* not applicable, *P* pathogenic, *VUS* variant of unclear significance.

^a^Paternity not confirmed.

^b^Apparently non-consanguineous families.

^c^*FBN1* mRNA reference number: NM_000138.4.

^d^*SKI* mRNA reference number: NM_003036.3.

^e^*TGFBR2* mRNA reference number: NM_001024847.2.

### Clinical findings in 74 individuals with a molecular diagnosis

#### Marfan syndrome

All patients with MFS and a rare *FBN1* variant (including c.2419 + 3delinsTTTTAGATCCATATTTTAG interpreted as VUS) met the revised Ghent criteria. Familial occurrence was observed in 19/36 families (52%; CI 95% 37–68). Detailed phenotypic information is provided in Table 2. Aortic root dilatation (z-score > 2) was noted in 77% (CI 95% 65–86) followed by mitral valve prolapse in 67% (CI 95% 54–78) and tricuspid valve prolapse in 53% (CI 95% 41–66) of patients. We also noted atrial septal defect, pulmonary artery dilatation and cardiomegaly in three individuals each. Six patients underwent aortic or mitral valve replacement in view of valve insufficiency. We observed myopia in 60% (CI 95% 47–72) followed by lens subluxation in 49% (CI 95% 36–62), dolichostenomelia in 71% (CI 95% 58–82) and pectus abnormality in 47% (CI 95% 34–60) of individuals. In addition, there was hypotonia in seven individuals, truncal obesity in three and developmental delay or mild intellectual disability in two individuals. Developmental delay or intellectual disability was however not investigated further.

**Table 2:** Clinical features in patients with Marfan or Loeys–Dietz syndrome and a clinically relevant variant.

Features	Marfan syndrome (n = 63)	Loeys–Dietz syndrome (n = 9)
**Ocular manifestations**		
Myopia	32/53 (60%)	1/8 (12%)
Ectopia lentis	26/53 (49%)	0/8
Early cataract	05/53 (9%)	0/8
Astigmatism	03/53 (5%)	2/8 (25%)
Microspherophakia	02/53 (3%)	0/8
**Cardiovascular manifestations**		
Aortic root dilatation	45/58 (77%)	6/8 (75%)
Aortic regurgitation	14/58 (24%)	3/8 (37%)
Aortic aneurysm^a^	04/58 (6%)	0/8
Aortic dissection	01/58 (1.7%)	1/8 (12%)
Mitral valve prolapse	39/58 (67%)	5/8 (62%)
Tricuspid valve prolapse	31/58 (53%)	5/8 (62%)
Mitral regurgitation	33/58 (56%)	4/8 (50%)
Tricuspid regurgitation	26/58 (44%)	3/8 (37%)
Bicuspid aortic valve	01/58 (1.7%)	1/8 (12%)
**Skeletal findings**		
Pectus abnormality	25/53 (47%)	7/8 (87%)
Scoliosis	16/53 (30%)	3/8 (37%)
Thumb sign	36/51 (70%)	3/8 (37%)
Wrist sign	35/51 (68%)	2/8 (25%)
Dolichostenomelia	38/53 (71%)	2/8 (25%)
Pes planus	27/53 (50%)	5/8 (62%)
Talipes deformity	13/53 (24%)	3/8 (37%)
Genu valgum/recurvatum	05/53 (9%)	1/8 (12%)
Reduced elbow extension	07/53 (13%)	0/8
Camptodactyly	19/53 (35%)	1/8 (12%)
Long and narrow feet	33/53 (62%)	5/8 (62%)
Metatarsus adductus	07/53 (13%)	1/8 (12%)
Craniosynostosis	0/53	2/8 (25%)
**Facial features**		
Long and narrow face	36/53 (67%)	8/8 (100%)
High arched palate	34/53 (64%)	5/8 (62%)
Bifid uvula/cleft palate	0/53	3/8 (37%)
Hypertelorism	01/53 (5%)	8/8 (100%)
Exotropia	05/53 (9%)	0/8
Dolichocephaly	10/53 (18%)	2/8 (25%)
Enophthalmos	15/53 (28%)	0/8
Downslanted palpebral fissures	19/53 (35%)	8/8 (100%)
Malar hypoplasia	31/53 (58%)	4/8 (50%)
Micro/retrognathia	18/53 (33%)	8/8 (100%)
Low-set ears	16/53 (30%)	2/8 (25%)
Crowding of teeth	08/53 (15%)	2/8 (25%)
**Other features**		
Skin striae	11/53 (20%)	2/8 (25%)
Skin laxity	06/53 (11%)	1/8 (12%)
Pneumothorax	06/53 (11%)	0/8
Dural ectasia	Not tested	Not tested
Hernia	07/53 (13%)	3/8 (37%)
Joint laxity	11/53 (20%)	4/8 (50%)
Translucent skin	06/53 (11%)	0/8
Decreased muscle mass	16/53 (30%)	4/8 (50%)
Joint contractures	02/53 (3%)	1/8 (12%)
Developmental delay/mild intellectual disability/motor delay	02/53 (3%)	4/8 (50%)

Numerator indicates the number of individuals with a clinical feature and denominator indicates the number of individuals where information is available for the given clinical feature.

^a^Younger individuals with Loeys–Dietz syndrome might not manifest with aortic aneurysm.

**Figure 2:**
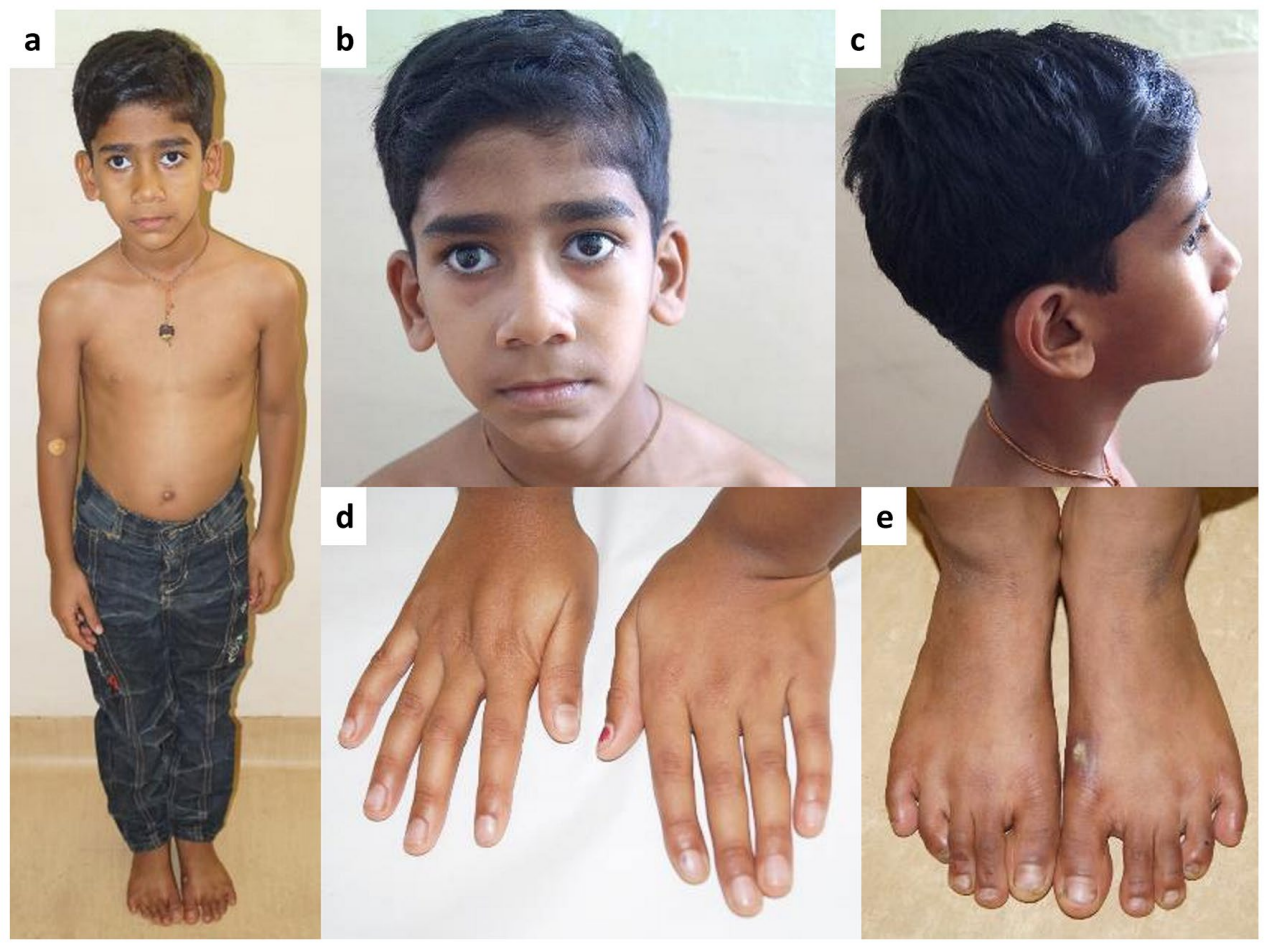
Photographs of patient 10 with the homozygous variant c.2861G > A, p.(Arg954His) in *FBN1*. He had normal stature (0.01 z) at 5 years 8 months of age with long face, exotropia of right eye, thin vermilion of upper lip (**a**–**c**), normal fingers (**d**) and pes planus (**e**).

In a 5-years-8-months-old male (patient 10) we identified the homozygous pathogenic *FBN1* missense variant c.2861G > A/p.(Arg954His) (Supplementary Table S1). He is the first child of a third degree consanguineous couple. His measurements were: weight of 20 kg (− 0.30 z), height of 116.5 cm (0.01 z) and head circumference of 49.5 cm (− 1.94 z). He had a long face, exotropia of the right eye, thin vermilion of the upper lip, high arched palate, bilateral lens subluxation, pes planus, mild distal joint laxity, bicuspid aortic valve, tricuspid and mitral valve prolapse and aortic sinus z-score of 2.86 (Fig. 2). Limited clinical information could be gathered via a video consultation and we specifically noted absence of breathlessness, visual problems, skin striae and chest deformity in parents. Mother however had features suggestive of Leri–Weill dyschondrosteosis (short stature, Madelung deformity, with similarly affected females and mildly affected males in the family).

#### Loeys–Dietz syndrome (LDS)

**Figure 3:**
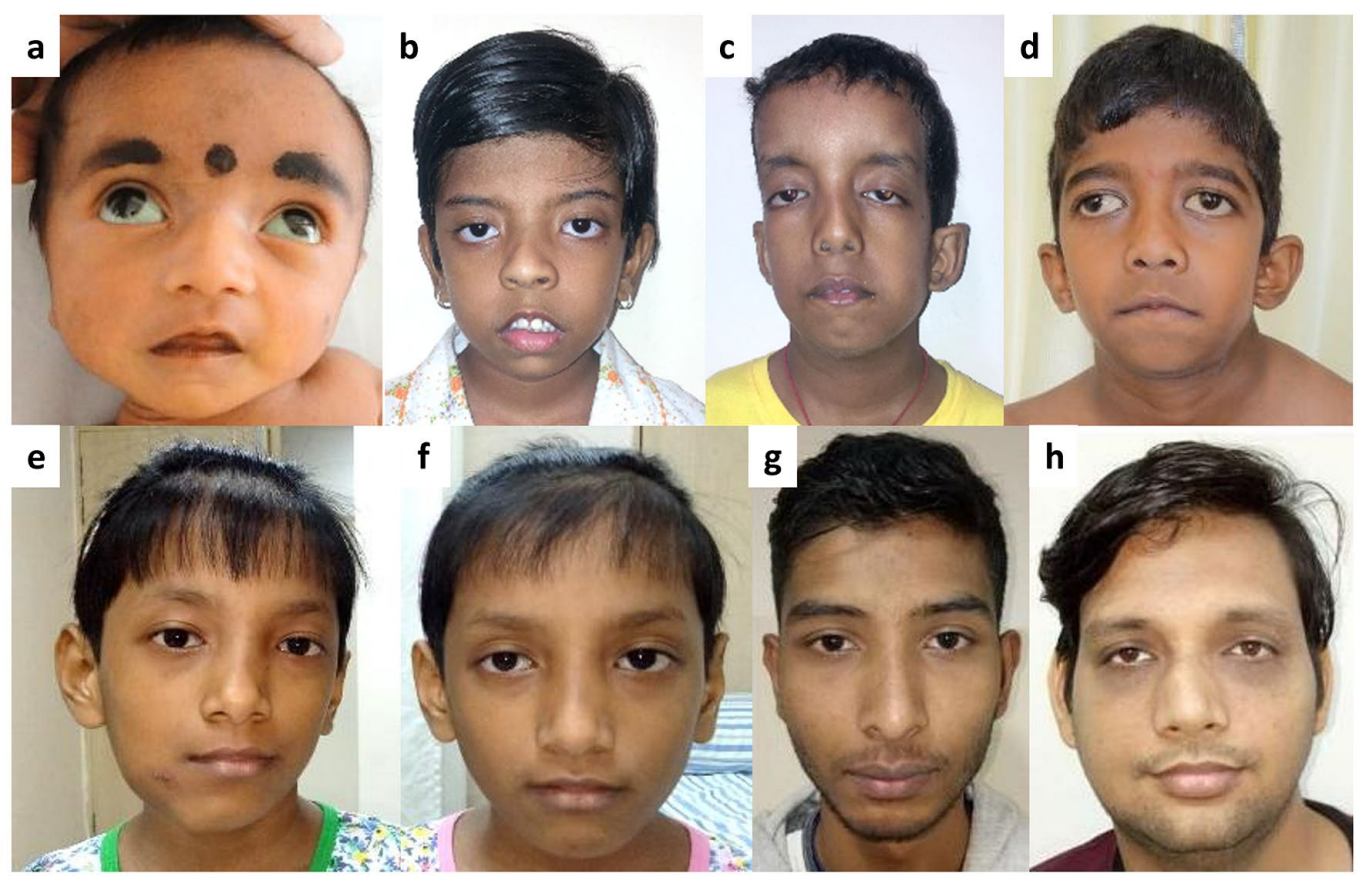
Facial photographs of patients with LDS show long faces, widely spaced eyes, downslanted palpebral fissures, thin vermilion of upper lips and micrognathia in all of them (**a**–**h**). Additionally, low set ears (**b**–**d**), ptosis (**b**) and wide mouth with downturned corners (**d**) were noted. Photographs were taken at ages: 7 months (**a**: Patient 44), 6 years (**b**: Patient 39 and **c**: Patient 40), 8 years (**d**: Patient 42), 9 years (**e**: Patient 45 and **f**: monozygotic twin of Patient 45), 16 years (**g**: Patient 41) and 36 years (**h**: Patient 43).

Nine patients from seven families (7/53, 13%; CI 95% 7–25) tested positive for a *TGFBR1* or *TGFBR2* rare variant and clinically presented with LDS. Phenotypic features are summarized in Table 2. Cardiac manifestation was observed in all individuals with aortic root dilatation in six individuals (6/8, 75%; CI 95% 41–93). One proband had a dissection of the aorta at 34 years of age. Typical facial features such as long and narrow face, hypertelorism, downslanted palpebral fissures and micro/retrognathia were seen in all eight patients (Table 2, Fig. 3). Three (37%; CI 95% 14–69) individuals had cleft palate/bifid uvula. Additionally, we noted developmental delay or motor delay (four patients), craniosysostosis (two patients), atopic dermatitis and anemia (in monozygotic twins), platybasia with basilar invagination and atlantoaxial subluxation with retroflexion (one individual) and joint dislocation (one individual) in patients with LDS.

#### Shprintzen–Goldberg syndrome (SGS)

**Figure 4:**
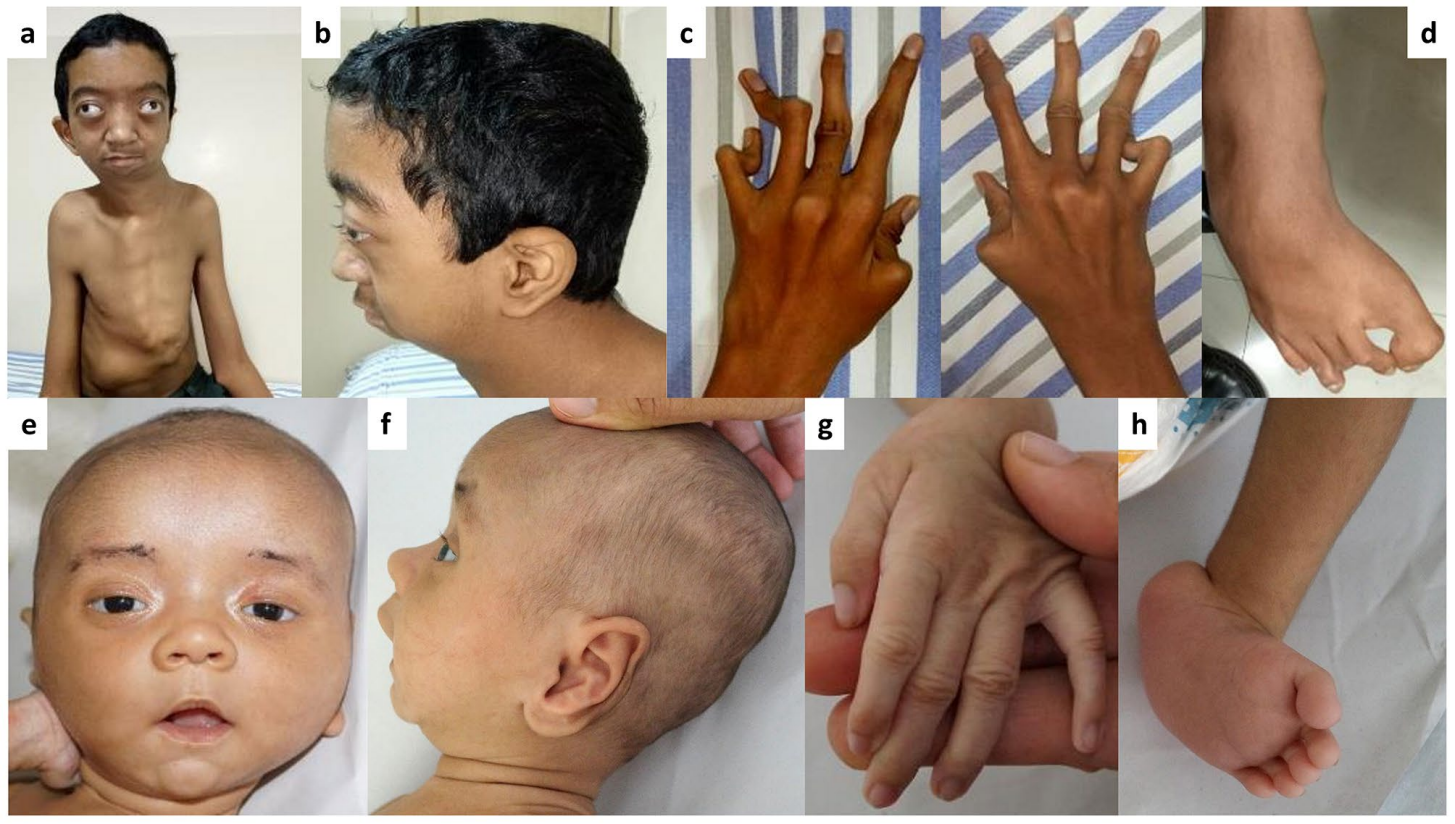
Clinical photographs of patients with SGS. Craniosynostosis, dolichostenomelia, low-set ears, overfolded ear helix, proptosis, downslanted palpebral fissures, hypertelorism, proptosis, alternative exotropia, vertical strabismus of left eye, microcornea, depressed nasal bridge, underdeveloped cheekbone, thin vermilion of upper lip, micro-retrognathia, pectus carinatum and kypho-scoliosis can be noted in patient 37 at age 13.5 years (**a**, **b**). He also had long and narrow fingers with camptodactyly (**c**), long and narrow feet with camptodactyly, pes planus and metatarsus adductus (**d**). The second patient (Patient 38), at 3 months of age, shows wide anterior fontanel, long ears, hairy pinnae, excess scalp skin, short and downslanted palpebral fissures, hypertelorism, depressed nasal bridge, inverted V shaped upper lip with think vermilion (**e**, **f**), long and narrow fingers (**g**) and bilateral talipes equinovarus (**h**).

Patient 37 aged 13.5 years had height of 154 cm (− 1.09 z), weight of 35 kg (− 1.91 z) and head circumference of 53 cm (− 1.49 z). Craniosynostosis, dolichostenomelia, low-set ears, overfolded ear helix, proptosis, downslanted palpebral fissures, hypertelorism, alternative exotropia, vertical strabismus of the left eye, microcornea, depressed nasal bridge, malar flattening, thin vermilion of upper lip, micro-retrognathia, high arched palate and malocclusion of teeth were noted in him (Fig. 4a,b). He also had pectus carinatum, kyphoscoliosis, long and narrow fingers, decreased palmar creases, long and narrow feet, camptodactyly of fingers and toes, pes planus, metatarsus adductus, recurrent or incisional hernia and decreased muscle mass (Fig. 4c,d). Echocardiography revealed myxomatous prolapsing atrioventricular valves with tricuspid and mitral regurgitation and aortic root dilatation (z-score 6.7). Computed tomography of skull showed calvarial thickening in fronto-parietal bones with partially fused coronal and sagittal sutures. Anterior displacement of the atlas from the occipital condyle and atlanto-occiptal assimilation, mild levoscoliosis of the cerivothoracic vertebra and mild dextroscoliosis of the thoracic vertebra (T9) were observed on computed tomography of the spine.

The second patient (patient 38), at 3 months of age, weighed 4.3 kg (− 2.29 z), had a length of 61 cm (− 0.29 z) and head circumference of 39.5 cm (− 1.58 z). His mother had gestational diabetes mellitus. He had wide anterior fontanel, long ears, hairy pinnae, short and downslanted palpebral fissures, hypertelorism, depressed nasal bridge, inverted V-shaped upper lip with thin vermilion, high arched palate, bifid uvula, excess scalp skin, long and narrow fingers, bilateral talipes equinovarus and Mongolian spots (Fig. 4e–h). Mild pectus excavatum, skin laxity, cutis marmorata and umbilical hernia were also noted in him. Echocardiography was normal with z-score of 0.2 for the aortic root size.

### Clinical findings in 8 families without a clinically relevant variant in 62 HCTD-related genes

Eight families (15%; CI 95% 8–27) did not have a clinically significant variant in 62 HCTD-related genes (disease genes and candidate genes). Clinical features of the eight index patients (46–53 and the brother of patient 52) are summarized in Table 3. None of them met the revised Ghent criteria. However, cardiac abnormalities were noted in all except one, and systemic score ≥ 7 was observed in two individuals. About half of them (4/9) fit criteria of the MASS (Mitral valve, Aorta, Skin, and Skeletal features) phenotype (MIM#604308), followed by Mitral valve prolapse syndrome (MIM%157700; #607829; %610840) (2/9), aortopathy (2/9) and an individual with Marfan-like disorder.

**Table 3:** Patients with no clinically relevant variant in genes known to cause Marfan syndrome and related disorders.

Patient #	Age at evaluation (years)	Gender	Revised Ghent criteria	Cardiac/vascular manifestations	Ocular features	Systemic score	Clinical diagnosis (pre-test)
46	15	Male	Negative	Tricuspid and mitral valve prolapse with regurgitation	Myopia	Negative	MASS phenotype
47^a^	15	Female	Negative	Tricuspid and mitral valve prolapse with regurgitations	Absent	Negative	Mitral valve prolapse syndrome
48	22	Male	Negative	Thoracic and abdominal aortic aneurysm	Absent	Negative	Aortopathy
49	16	Female	Negative	Mitral valve prolapse regurgitation	Absent	Negative	MASS phenotype
50	13	Female	Negative	Myxomatous mitral valve with regurgitation	Absent	Negative	Mitral valve prolapse syndrome
51	18	Male	Negative	Myxomatous mitral valve with regurgitation	Absent	Positive	MASS phenotype
52^b,c^	2	Male	Negative	Mild aortic root dilatation with ventricular septal defect	Absent	Negative	Aortopathy with facial dysmorphism
52’s brother^c^	2	Male	Negative	Absent	Absent	Negative	MASS phenotype
53^d^	15	Female	Negative	Arteritis	Absent	Positive	Marfanoid disorder

Revised Ghent criteria ‘negative’ indicates non-fulfillment. Systemic score ‘positive’ indicates systemic involvement (score ≥ 7) and ‘negative’ suggests no systemic involvement (score < 7).

*MASS phenotype* Mitral valve, Aorta, Skin, and Skeletal features.

^a^Patient 47 had poor scholastic performance.

^b^Patient 52 had developmental delay.

^c^The difference in the phenotypes of patient 52 and his brother could suggest variable expression.

^d^Patient 53 had microtia and perauricular tag.

## Discussion

We describe the clinical spectrum and genetic findings in 83 individuals from 53 Indian families with MFS, LDS and SGS. This is so far the largest cohort of Indian patients with a definitive molecular diagnosis for an aortopathy in a total of 45 index patients. The identification of clinically significant variants in MFS and related disorders reduces the uncertainty in diagnosis in individuals with a suspected diagnosis and guides appropriate management of their cardiovascular and ocular complications. Our study also provides the mutation spectrum in Indian patients with these three types of HCTDs and adds 21 novel rare variants.

We could not find any published reports on Indian patients with LDS or SGS with a molecular diagnosis. Our report now adds seven patients with LDS, two novel *TGFBR2* variants, two patients with SGS and a novel disease-causing variant in the R-SMAD binding domain of *SKI* to the literature. We did not note any unusual clinical features in our small cohort of individuals with LDS and SGS.

Previously, only two publications have reported pathogenic variants in *FBN1* in individuals from India^28,29^. One reported a fetus with arthrogryposis, multiple joint dislocations, scoliosis and facial dysmorphism who carried the variant p.(Pro2002Ser). Incidentally the fetus had a variant in *FBN2* too (NM_001999.3:c.2945G > T; p.(Cys982Phe)). However, segregation of the variants in the family was not performed^28^. The second family comprised 27 individuals with ectopia lentis in whom the *FBN1* missense variant p.(Arg240Cys) segregated^29^. Absence of other cardinal manifestations of MFS suggests occurrence of autosomal dominant “isolated” ectopia lentis 1 in the family (MIM#129600).

Several large cohorts on MFS and related disorders have been published previously with patients originating from European countries or China^23,30–36^. They report a definitive molecular diagnosis in 40–95% of individuals, depending on the inclusion criteria and the testing strategy. We obtained a molecular diagnosis in 45/53 (85%; CI 95% 73–92) families by NGS, including targeted multiple gene panel and whole-exome sequencing, and multiplex ligation-dependent probe amplification.

In our cohort, 36 of the 45 (80.0%; CI 95% 66–89) patients carried a rare variant in *FBN1*, and the majority of them were missense (21, 58.3%; CI 95% 42–73) in concordance with the literature^34,37^, and 13 substituted or introduced a cysteine residue. Also, we observed splicing variants and multi-exon deletions (five each or 13.9% each; CI 95% 6–29 each), small deletion/insertion (three, 8.3%; CI 95% 3–22) and nonsense (two, 5.6%; CI 95% 2–18) variants in *FBN1*. In the literature only ~ 5% of probands with an *FBN1* pathogenic variant have been reported to carry a deletion or duplication^2,33^, which is 2.8-fold lower in our relatively small cohort of Indian patients. We noted missense variants in four (8.9%; CI 95% 4–21) patients in *TGFBR1,* three (6.7%; CI 95% 2–18) in *TGFBR2*, and two (4.4%; CI 95% 1–15) in *SKI.* Overall, there were 41 rare variants in four genes, with four variants identified in more than one family indicating the private nature of the remaining variants^37^. 51.2% (CI 95% 36–66) of the rare variants were novel, which is similar to the percentages reported in other studies (46.6–67.5%)^19,23,26,31,32,34^.

Disease-causing variants in exon 24–32 have been associated with early-onset and rapidly progressive MFS^2,30,38^. Seven (7/36) index patients of our cohort have pathogenic variants in this region of the *FBN1* gene. Their age at diagnosis ranged from 4 months to 7 years except one (23-years-old). Four of them had de novo variants whereas three were familial. We also report on a proband (patient 11) and his paternal half-sister with the *FBN1* nonsense variant c.3012C > A/p.(Tyr1004*). The father did not carry this variant in leukocyte-derived DNA indicating germline mosaicism in him. Five of the seven (71%; CI 95% 36–92) had ocular, cardiac and skeletal manifestations. We also had a 4-months-old infant with early onset MFS in our cohort. She had atrioventricular valve prolapse with severe mitral regurgitation, ostium secundum type of atrial septal defect measuring 11 mm, dilated chambers of heart with dilated aortic root, scoliosis, skin laxity and long and narrow fingers. She succumbed to cardiac failure at 6 months of age.

Bi-allelic *FBN1* variants have been reported in 16 families (eight with homozygous and eight with compound heterozygous variants) with MFS^39–45^. We also document a patient (patient 10) with the homozygous *FBN1* missense variant c.2861G > A/p.(Arg954His). Similar to the present individual, all the families reported in the literature with homozygous variants were consanguineous, except one (7/8, 87.5%; CI 95% 53–98). The initially described seven patients with bi-allelic variants had a severe clinical course with early age of onset ranging from day 7 to 22 years^39–42,44,45^. However, Arnaud et al. reported nine families with bi-allelic variants in *FBN1* with classical and mild clinical features with age at diagnosis ranging from 8 to 53 years^43^. In the 16 reported families with bi-allelic *FBN1* variants 17 missense variants, two frameshift and one nonsense variant have been identified^43^. Together with the p.(Arg954His) variant detected in patient 10 reported here, the vast majority of bi-allelic variants represent amino acid substitutions (18/21; 85.7%; CI 95% 65–95). Patient 10 at 5-years-8-months presented with typical facial features, bilateral ectopia lentis, bicuspid aortic valve with z-score of ≥ 2 and atrioventricular valve prolapse, the classical form of MFS. Although we were unable to perform a detailed clinical examination of patient 10’s parents who are heterozygous carriers of the p.(Arg954His) variant, the same heterozygous variant has been previously reported in a 58-year-old female with skeletal features, ectopia lentis but no cardiovascular abnormalities^27^. In the gnomAD browser, the variant was listed in 1 out of 251,154 alleles. Heterozygous carriers of the p.(Arg2726Trp) variant, who have a second pathogenic *FBN1* variant on the other allele in three families, only had isolated skeletal features typical of MFS and/or high stature^43^. In addition, incomplete penetrance has been reported for individuals carrying the p.(Arg2726Trp) variant in the heterozygous state^46^, which is in line with a worldwide minor allele frequency (MAF) of 0.067% for this *FBN1* variant (gnomAD browser). Interestingly, a worldwide MAF of 0.02% and 0.12% (gnomAD browser) for the *FBN1* alterations p.(Pro1424Ala) and p.(Ala986Thr)^43^, respectively, also suggests incomplete penetrance in individuals carrying either of the variants in the heterozygous state and full penetrance in individuals with one of the two aforementioned *FBN1* variants *in trans* with a second pathogenic variant. Although further studies are needed to study the effect of recessive *FBN1* missense variants on fibrillin-1 function, several of the 18 missense variants identified in a homozygous or compound heterozygous state may act as hypomorphic alleles.

Eight families (15%; CI 95% 8–27) did not have a clinically significant variant in genes known to cause MFS or associated with HCTD (62 genes on NGS panel), and similar observations were reported in the literature^33,34^. Targeted panel NGS testing has considerable limitations in the detection of single- and multi-exon deletions/duplications and structural variants as well as non-coding and regulatory variants. Thus, clinically relevant variants might have been missed in one or several of the eight index patients. None of the eight patients met the revised Ghent criteria. The majority of the negative patients have atrio-ventricular valve prolapse with regurgitation. We observed poor scholastic performance (P47)/developmental delay (P52), ectopic and horseshoe kidney with polycystic ovaries (P50) and microtia and pre-auricular tag (P53) in some of them. Whole-exome or whole-genome sequencing will be performed in the eight families to identify the genetic cause underlying the disease in the index patients.

In conclusion, we describe the first and largest cohort of patients with MFS or related disorders from India and provide a base for further genetic testing in this large population. About half of them harbored a novel variant, which has expanded the mutation spectrum of these disorders. Biallelic *FBN1* missense variants can be present in individuals with classic MFS and may point to hypomorphic *FBN1* alleles manifesting only when present in the homozygous or compound heterozygous state. Identification of clinically significant variants reduces uncertainty in diagnosis in suspected individuals and guides appropriate management of their cardiovascular and ocular complications. Yet genetically unsolved patients with MFS-like conditions in this cohort suggests further genetic heterogeneity and the presence of phenocopies.

## Methods

### Study approval

The study was approved by the Institutional Ethics Committee, Kasturba Medical College and Hospital, Manipal (IEC No: 118/2016) and Narayana Health Academic Ethics Committee, Narayana Health Hospitals, Bangalore (NH/AEC-CL-2017-191). Informed consent for clinical data, samples and publication of photographs was obtained from parents/legal guardians of patients or the patients themselves. All experiments were performed in accordance with relevant guidelines and regulations.

### Patient cohort and data collection

We recruited pediatric, adolescent and adult patients referred for genetics counseling at Kasturba Hospital, Manipal, India and Narayana Hrudayalaya Hospitals, Bangalore, India with features suggestive of MFS, aortopathy or related HCTDs over a period of 5 years. Clinical data and samples for all individuals were obtained with informed consent of patients’ parents/legal guardians or the patients themselves, including written consent to use photographs in this report. Clinical data that included a three-generation pedigree and family history of similarly affected individuals (specifically for the presence of tall stature, ocular abnormalities or visual defects and cardiac surgeries) were noted. We performed physical examination and recorded anthropometry for all patients. We collected echocardiographic information and calculated z-score for the aortic root measurements. Ophthalmological evaluation comprised a slit-lamp examination. We performed radiographic assessment and other imaging whenever necessary. Revised Ghent criteria was used for the diagnosis of MFS^47,48^. We collected two millilitres of blood samples from patients and their available family members including parents and siblings for genomic DNA isolation.

The lower and upper limits of the 95% confidence interval (CI 95%) for a proportion were calculated with the VassarStats tool (http://vassarstats.net/index.html) according to the method previously described^49^.

### Molecular genetic analysis

Genomic DNA was isolated from leukocytes by standard procedures. For targeted NGS of the DNA sample of patients 1, 2, 5–8, 10–15, 17–24, 26, 28–37, 39–41, 43, and 45–53, we initially selected the coding region and adjacent intronic sequences of 18 genes (*ACTA2* (NM_001613.2), *BGN* (NM_001711.5), *CBS* (NM_000071.2, NM_001321072.1), *COL3A1* (NM_000090.3), *FBN1* (NM_000138.4), *FBN2* (NM_001999.3), *LOX* (NM_002317.6), *MFAP5* (NM_003480.3), *MYH11* (NM_001040113.1), *MYLK* (NM_053025.3), *NOTCH1* (NM_017617.4), *PRKG1* (NM_017617.4, NM_001098512.2), *SKI* (NM_003036.3), *SMAD3* (NM_005902.3), *TGFB2* (NM_001135599.2), *TGFB3* (NM_003239.3), *TGFBR1* (NM_004612.3), and *TGFBR2* (NM_001024847.2)) related to syndromic and non-syndromic forms of aortopathies and connective tissue disorders. Enrichment of the regions of interest (ROI) was performed with the Illumina Rapid Capture Custom Enrichment kit or the Illumina Nextera Flex for Enrichment kit according to the manufacturer’s instructions. Briefly, following fragmentation of genomic DNA, fragmented DNA was amplified and patient-specific (index) adapters were added by PCR. Samples from 12 patients were combined into one single hybridization mix containing target-specific capture probes. The DNA-probe hybrids were then captured with streptavidin beads, and non-targeted DNA fragments as well as unspecific binding were removed by heated washes. Next, the captured DNA library was eluted from the beads, purified and amplified by PCR. For generation of clusters and subsequent sequencing of the targeted DNA samples on a flow cell, a sequencing reagent kit from Illumina was used. High-throughput NGS data were generated on an Illumina sequencing platform^26^.

SALSA MLPA kits P065-C1 and P066-C1 Marfan Syndrome, P148-B3 TGFBR1-TGFBR2, and P155-D2 COL3A1 (MRC-Holland) were used according to the manufacturer’s instructions to detect single and multiple exon deletions/duplications in *FBN1* (all 66 exons), *TGFBR1* (all nine exons), *TGFBR2* (all eight exons) and *COL3A1* (exons 1, 2, 4, 5, 9, 11, 14, 17, 20, 23, 28, 36, 43, 47, and 51). PCR products were separated on an automated capillary DNA sequencer (ABI 3500; Applied Biosystems). MLPA data were analysed with the Sequence Pilot module MLPA software (JSI Medical Systems)^26^.

For patients 46–53 without a pathogenic variant in one of the aforementioned 18 genes, the analysis was extended to the 44 additional genes on the customized NGS panel [*ADAMTS10* (NM_030957.3), *ADAMTS2* (NM_014244.4), *B3GALT6* (NM_080605.3), *B4GALT7* (NM_007255.2), *CDKL1* (NM_004196.4), *CHST14* (NM_130468.3), *COL1A1* (NM_000088.3), *COL1A2* (NM_000089.3), *COL2A1* (NM_001844.4), *COL4A1* (NM_001845.5)*, COL4A5* (NM_000495.4), *COL5A1* (NM_000093.4), *COL5A2* (NM_000393.3), *DCHS1* (NM_003737.3), *DIDO1* (NM_033081.2), *DUOX2* (NM_014080.4), *EFEMP2* (NM_016938.4), *ELN* (NM_001278939.1), *EMILIN1* (NM_007046.3), *FBLN5* (NM_006329.3), *FKBP14* (NM_017946.3), *FLNA* (NM_001110556.1), *FLNC* (NM_001458.4), *FOXE3* (NM_012186.2), *FOXS1* (NM_004118.3), *GATA5* (NM_080473.4), *KDR* (NM_002253.2), *LRP1* (NM_002332.2), *LTBP2* (NM_000428.2), *LTBP4* (NM_003573.2), *MAT2A* (NM_005911.5), *PEAR1* (NM_001080471.1), *PLK1* (NM_005030.5), *PLOD1* (NM_000302.3), *PLOD3* (NM_001084.4), *PRDM5* (NM_018699.3), *SLC2A10* (NM_030777.3), *SLC39A13* (NM_152264.4), *SMAD2* (NM_005901.5), *SMAD4* (NM_005359.5), *SOX18* (NM_018419.2), *TNXB* (NM_019105.6), *ULK4* (NM_017886.3), *ZNF469* (NM_001127464.2)]. ROI sequences were aligned to the human reference genome (hg19) and visualized and evaluated by the Sequence Pilot module SeqNext software (JSI Medical Systems). NGS data of patients 46–53 were analysed for single nucleotide variants and copy number variations in all 62 panel genes.

Enrichment of the regions of interest for patients 3, 9, 25, 27, 38, 42 and 44 was performed with a custom Haloplex enrichment kit according to the manufacturer’s protocol (Agilent Technologies) as described previously^19^. Compared to the original kit described in Proost et al. (2015) the custom Haloplex enrichment kit contained additional probes for *PRKG1* (ENST00000401604), *TGFB3* (ENST00000238682), *MAT2A* (ENST00000306434) and *MFAP5* (ENST00000359478) for patients 3, 9, 25, 27, 38, 42 and 44, *FOXE3* (ENST00000335071) for patients 9, 25, 27, 38 and 44 and *ELN* (ENST00000358929), *FBN2* (ENST00000262464) and *SMAD2* (ENST00000402690) for patients 27 and 38. The concentration of each library was measured by Qubit fluorometric quantification (Life Technologies). For generation of clusters and subsequent sequencing of the targeted DNA samples on a flow cell, a sequencing reagent kit from Illumina was used. High-throughput NGS data were generated on an Illumina sequencing platform. ROI sequences were aligned to the human reference genome (hg19) and visualized and evaluated by the Sequence Pilot module SeqNext software (JSI Medical Systems)^19^.

Whole-exome sequencing (WES) in patients 4 and 16 was performed either with Nextera Rapid Capture Exomes (Illumina) or Agilent SureSelect V6 (Agilent Technologies) kit. Massively parallel sequencing was done on an Illumina NextSeq Platform. There was an average coverage depth of 110×, with ~ 94% of bases covered at > 20× and the data was analysed using an in-house pipeline based on Burrows-Wheeler Aligner (v0.7.15)^50^ and Genome Analysis Toolkit Best Practices pipeline (v3.6)^51^. We used ANNOVAR to annotate the variant call format (vcf) files^52,53^. We integrated annotated data with phenotypes catalogued in Online Mendelian Inheritance in Man, human phenotype ontology (HPO) terms, and allele frequency details from in-house variant database of 870 exomes of Indians. Rare variants were retrieved with minor allele frequency of < 1% in population databases [Exome Aggregation Consortium (ExAC) and gnomAD^54,55^] and our in-house data. Variants were prioritized for the phenotypes^56^.

Identified sequence variants have been searched in the following databases: HGMD Professional versions 2017.1-2019.2 (https://portal.biobase-international.com/hgmd/pro/start.php)^57,58^, UMD-FBN1 (http://www.umd.be/FBN1/)^37^, and gnomAD v2.1.1. (https://gnomad.broadinstitute.org/)^54^. Classification of novel variants as pathogenic variants, likely pathogenic variants and variants of unknown significance (VUS) was performed according to the American College of Medical Genetics and Genomics and the Association for Molecular Pathology standards and guidelines^59^, either with the help of VarSome (https://varsome.com/)^60^ or by manual application of the guidelines. The functional impact of novel variants was assessed by the pathogenicity prediction programs CADD (http://cadd.gs.washington.edu/score)^61^, REVEL (https://sites.google.com/site/revelgenomics/downloads)^62^, and M-CAP (http://bejerano.stanford.edu/MCAP/)^63^. Genetic tolerance at the affected amino acid position in the protein was predicted by MetaDome (https://stuart.radboudumc.nl/metadome/)^64^. Splice site prediction scores for novel intronic variants were calculated for wild-type and mutated sequences by using the in silico tools Human Splicing Finder 3.1 (http://umd.be/HSF3/HSF.shtml), NetGene2 (http://www.cbs.dtu.dk/services/NetGene2/), and the Berkeley Drosophila Genome Project Database (https://www.fruitfly.org/seq_tools/splice.html)^65–68^.

Sanger sequencing was performed for validation of pathogenic, likely pathogenic sequence variants and VUS identified by NGS and for regions of interest covered by less than 20 reads. Segregation analysis of pathogenic and likely pathogenic variants in affected and/or healthy family members of the index patient was performed by Sanger sequencing using an automated capillary DNA sequencer (ABI 3500; Applied Biosystems). Sequence electropherograms were analysed using the Sequence Pilot module SeqPatient software (JSI Medical Systems).

All novel variants were deposited in the LOVD Database, where they are available under the DB-ID numbers 0000667876 to 0000667897, 0000708485 and 0000708486.

## Supplementary Information

Supplementary Information.

## Data Availability

All data generated or analysed during this study are included in this published article (and its “Supplementary Information File”).
