# Supplementary material for: Clinically relevant variants in a large cohort of Indian patients with Marfan syndrome and related disorders identified by next-generation sequencing
Source: Sci Rep. 2021 Jan 12;11:764. doi: 10.1038/s41598-020-80755-7 (PMC7804850; doi:10.1038/s41598-020-80755-7)
Supplement: Supplementary file 1 — Supplementary Information. [file 41598_2020_80755_MOESM1_ESM.docx]

**Clinically relevant variants in a large cohort of Indian patients with Marfan syndrome and related disorders identified by next-generation sequencing**

Shalini S Nayak, Pauline E Schneeberger, Siddaramappa J Patil, Karegowda M Arun, Pujar V Suresh, Viralam S Kiran, Sateesh Siddaiah, Shreesha Maiya, Shrikanth K Venkatachalagupta, Neethukrishna Kausthubham, Fanny Kortüm, Isabella Rau, Alexandra Wey-Fabrizius, Lotte Van Den Heuvel, Josephina Meester, Lut Van Laer, Anju Shukla, Bart Loeys, Katta M Girisha, Kerstin Kutsche

**Table S1.** Previously reported pathogenic variants in the genes *FBN1*, *SKI, TGFBR1*, and *TGFBR2,* clinical diagnosis in the index patients, and number of affected family members carrying the familial pathogenic variant

| **Gene** | **Patient #** | **Affected family members** | **Nucleotide change** | **Affected exon(s)** | **Amino acid alteration** | **HGMD**  **accession number, UMD-FBN1-ID or reference** |
| --- | --- | --- | --- | --- | --- | --- |
| ***FBN1* ^d^** | 2 | 2 | c.199T>T | Exon 3 | p.(Cys67Arg) | CM096435 |
|  | 7 | 1  (*de novo*) ^a^ | c.1792T>C | Exon 15 | p.(Cys598Arg) | CM180966 |
|  | 10 | 3 ^b^ | c.2861G>A | Exon 25 | p.(Arg954His) | CM091207 |
|  | 11 | 2 | c.3012C>A | Exon 25 | p.(Tyr1004*) | CM154826 |
|  | 12 ^c^ | 2 | c.3037G>A | Exon 25 | p.(Gly1013Arg) | CM950441 |
|  | 13 ^c^ | 1 | c.3037G>A | Exon 25 | p.(Gly1013Arg) | CM950441 |
|  | 15 | 1  (*de novo*) ^a^ | c.3599A>G | Exon 30 | p.(Glu1200Gly) | CM990590 |
|  | 17 | 1 | c.(3964+1_3965-1)_(4747+1_4748-1)del | Exons 33-38 | p.? | UMD-FBN1-ID 3248 |
|  | 19 | 4 | c.4615C>T | Exon 38 | p.(Arg1539*) | CM010044 |
|  | 20 | 1  (*de novo*) ^a^ | c.4781delG | Exon 39 | p.(Gly1594Valfs*46) | CD1915156 |
|  | 23 | 1  (*de novo*) ^a^ | c.5503T>G | Exon 45 | p.(Cys1835Gly) | CM1715580 |
|  | 24 | 1  (*de novo*) ^a^ | c.5578T>C | Exon 46 | p.(Cys1860Arg) | CM085406 |
|  | 26 | 3 | c.5671G>A | Exon 46 | p.(Asp1891Asn) | CM196595 |
|  | 31 | 2 | c.7039_7040delAT | Exon 58 | p.(Met2347Valfs*19) | CD020234 |
|  | 33 | 1 | c.7408T>A | Exon 60 | p.(Cys2470Ser) | Same amino acid substitution reported in Takeda *et al*. (2018)^1^ |
|  | 35 ^c^ | 3 | c.7828G>A | Exon 64 | p.(Glu2610Lys) | CM972822 |
|  | 36 ^c^ | 1  (*de novo*) ^a^ | c.7828G>A | Exon 64 | p.(Glu2610Lys) | CM972822 |
| ***SKI* ^e^** | 37 | 1 | c.100G>A | Exon 1 | p.(Gly34Ser) | CM129801 |
| ***TGFBR1* ^f^** | 39 ^c^ | 1  (*de novo*) ^a^ | c.722C>T | Exon 4 | p.(Ser241Leu) | CM061221 |
|  | 40 ^c^ | 1  (*de novo*) ^a^ | c.722C>T | Exon 4 | p.(Ser241Leu) | CM061221 |
|  | 41 | 2 | c.673C>T | Exon 4 | p.(Arg225Trp) | CM1916456 |
|  | 42 | 1  (*de novo*) ^a^ | c.1198G>A | Exon 7 | p.(Asp400Asn) | CM139800 |
| ***TGFBR2* ^g^** | 45 | 2 ^h^  (*de novo*) ^a^ | c.1657C>T | Exon 8 | p.(Arg553Cys) | CM050761 |

^a^ Paternity not confirmed.

^b^ Patient is homozygous for the variant, and parents are heterozygous carriers and consanguineous.

^c^ Apparently non-consanguineous families.

^d^ *FBN1* mRNA reference number: NM_000138.4.

^e^ *SKI* mRNA reference number: NM_003036.3.

^f^ *TGFBR1* mRNA reference number: NM_004612.3.

^g^ *TGFBR2* mRNA reference number: NM_001024847.2.

^h^ Two affected monozygotic twins.

HGMD, The Human Gene Mutation Database; UMB-FBN1, The Universal FBN1 Mutations Database.

**Reference:**

1. Takeda, N. *et al.* Impact of Pathogenic FBN1 Variant Types on the Progression of Aortic Disease in Patients With Marfan Syndrome. *Circ Genom Precis Med.* **11**, e002058, doi:10.1161/circgen.117.002058 (2018).
